# Giant Aneurysm of the Left Main Coronary Artery: A Case Report

**DOI:** 10.7759/cureus.94821

**Published:** 2025-10-17

**Authors:** Istria Barros, Alberto Navarro, Emilio Herrera, Juan Senior

**Affiliations:** 1 Epidemiology, Universidad Del Rosario, Bogota, COL; 2 Cardiology, Hospital Universitario San Vicente Fundación, Medellin, COL

**Keywords:** acute myocardial infarction, chest pain, coronary aneurysm, left main coronary artery, revascularization

## Abstract

Giant coronary artery aneurysm (CAA) is a rare and often underreported condition, most frequently associated with atherosclerosis but also linked to congenital, inflammatory, and connective tissue disorders. Unlike typical atherosclerotic disease, there is limited evidence regarding the most effective diagnostic tools, treatment options, and prognostic implications for patients with giant CAAs.

The left main coronary artery (LMCA) is an especially uncommon location, where management decisions can be particularly complex due to the lack of established guidelines. We present the case of a young patient with a giant aneurysm of the LMCA who was successfully treated with conservative medical therapy and remained asymptomatic after six months of follow-up without surgical or percutaneous intervention. This report highlights the challenges in choosing appropriate management strategies for giant CAAs and emphasizes the importance of individualized, patient-centered care. It also underscores the need for further studies to develop evidence-based recommendations for diagnosing and treating this rare but clinically significant condition.

## Introduction

Coronary artery aneurysm (CAA) is considered an intrinsic anomaly of coronary artery anatomy. It may be either acquired or congenital [1]. CAA is defined as a focal dilation measuring at least 1.5 times the diameter of the adjacent normal segment and may present with either a saccular or fusiform morphology. It is classified as *giant* when the diameter exceeds 20 mm. CAA occurs more frequently in men than in women, with a 3:1 ratio, and most commonly affects the right coronary artery (RCA), followed by the left anterior descending artery (LAD), and least widely the left main coronary artery (LMCA) [2].

The clinical presentation of CAA varies and may be asymptomatic, present as stable angina, or lead to an acute coronary syndrome, depending on the presence of in situ thrombosis, distal embolization, or rupture [3]. Coronary aneurysms are most commonly diagnosed through coronary angiography, with rare instances identified at autopsy [4].

We present the case of a 45-year-old man who experienced an anterior ST-elevation myocardial infarction (STEMI). He was initially treated with thrombolysis and subsequently underwent coronary angiography, which revealed a giant saccular aneurysm in the LMCA.

## Case presentation

**Figure 1:**
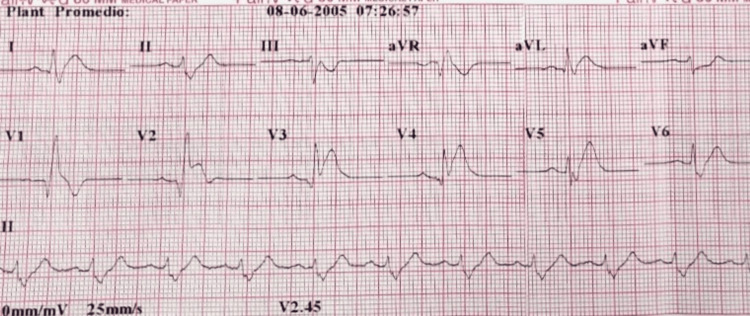
ECG: Anterior ST-elevation myocardial infarction (STEMI) with right bundle branch block. ECG, electrocardiogram

A 45-year-old man with a medical history of hypertension, dyslipidemia, and a paternal history of early sudden death of unknown cause was referred from a rural hospital due to an anterior STEMI with right bundle branch block on electrocardiogram (ECG) (Figure 1). He received thrombolysis with alteplase three hours after the onset of chest pain.

On physical examination, he had a heart rate of 85 beats per minute (bpm), blood pressure of 140/80 mmHg, a respiratory rate of 20 breaths/minute, and a temperature of 37.1 °C. Jugular venous pressure, cardiac, and pulmonary auscultation were normal. Abdominal exam, peripheral pulses, and neurological assessment were unremarkable. A follow-up ECG compared to the initial one showed a 50% reduction in ST-segment elevation, and there was no chest pain. Chest X-ray was normal.

**Figure 2:**
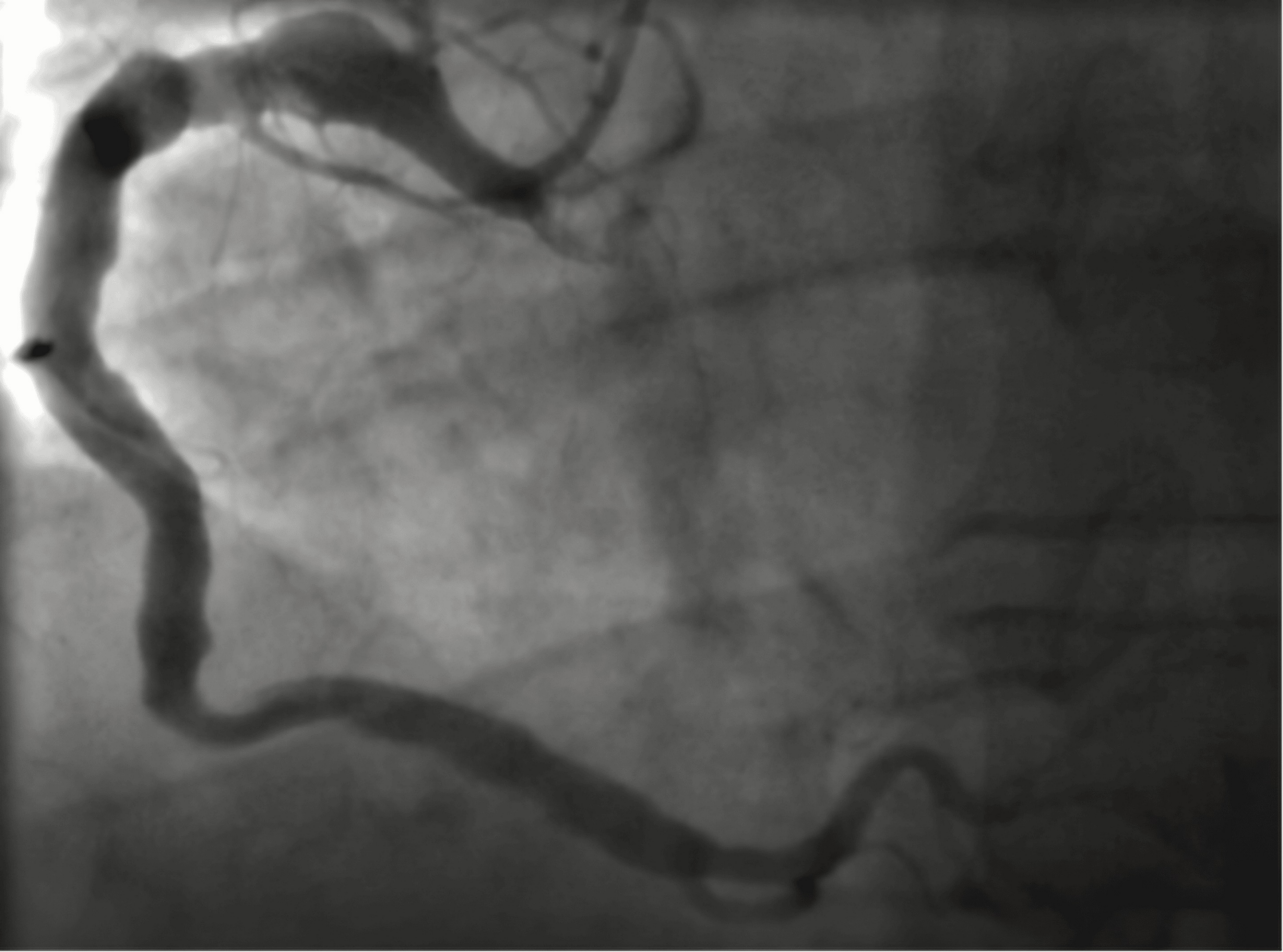
Right coronary artery ectasia.

**Figure 3:**
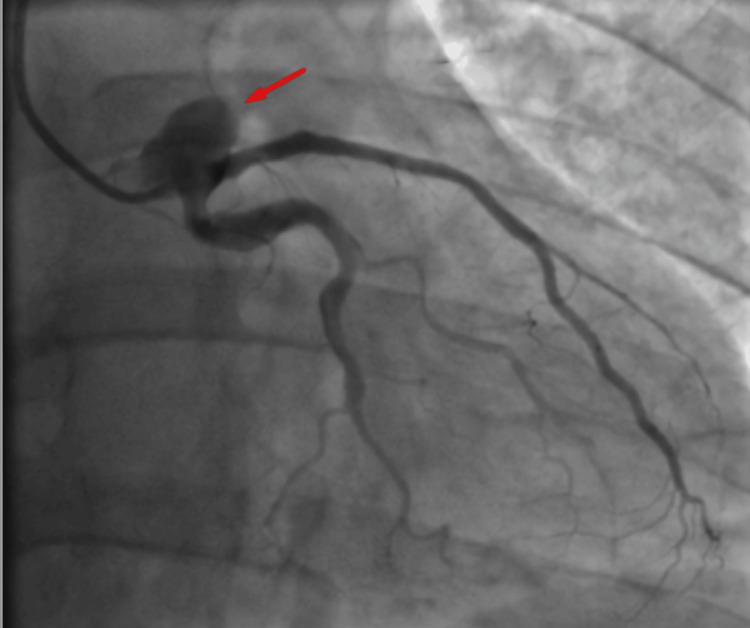
Giant aneurysm (arrow) of the left main coronary artery extending into the left anterior descending artery.

A coronary angiography was performed nine hours after symptom onset as part of a pharmacoinvasive strategy, revealing a dominant right coronary artery (RCA) with normal origin and diffuse ectasia (Figure 2), and a large-caliber LMCA with a giant aneurysm extending into the distal LMCA, proximal to the LAD artery (Figure 3). The proximal LAD also exhibited a giant aneurysm with preserved distal flow. The left circumflex artery and ramus intermedius had no critical lesions.

**Figure 4:**
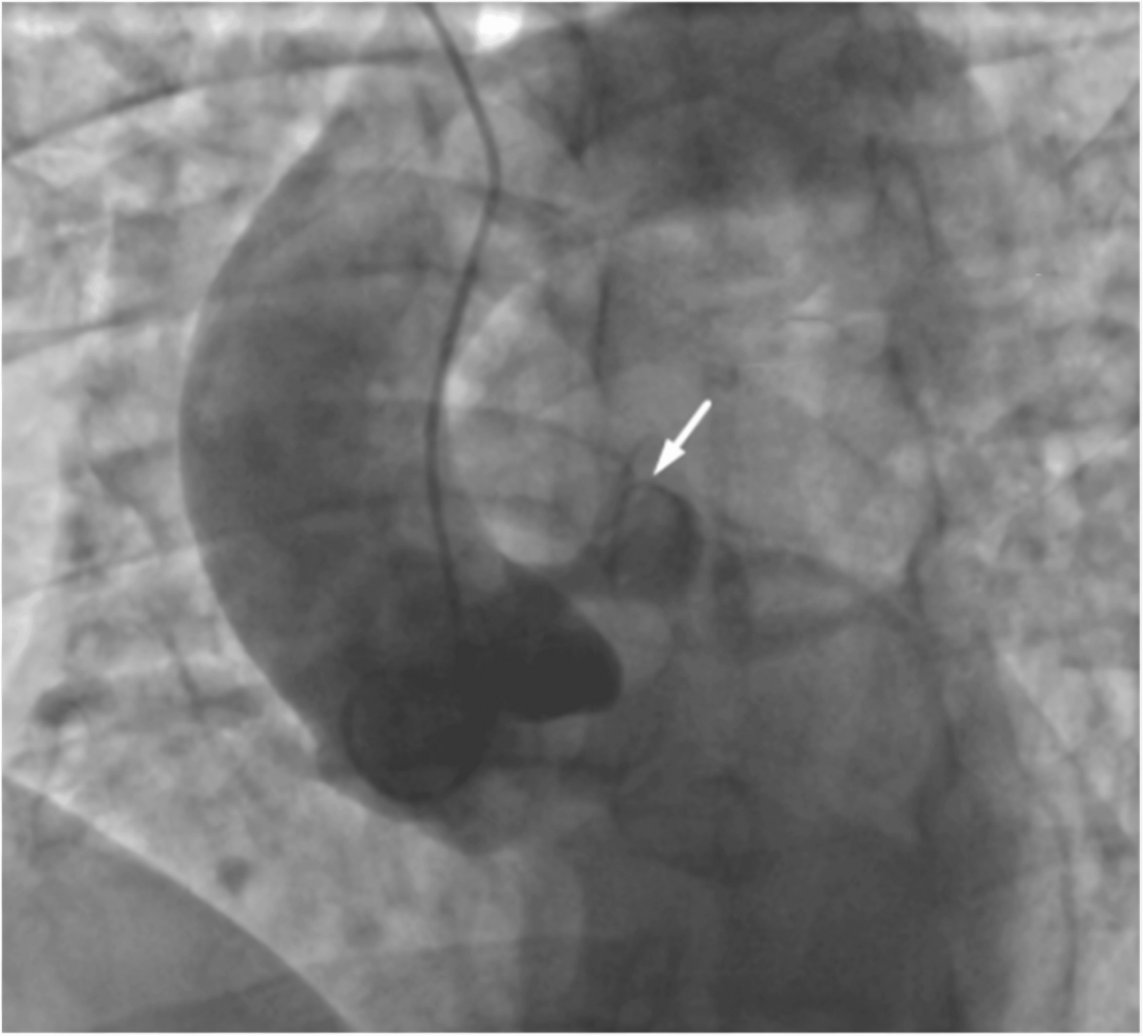
Thoracic aortogram showing a large aneurysm of the left main coronary artery with a filling defect suggestive of residual thrombus. The arrow shows the large aneurysmal dilation of the left main coronary artery with a filling defect consistent with residual thrombus.

**Figure 5:**
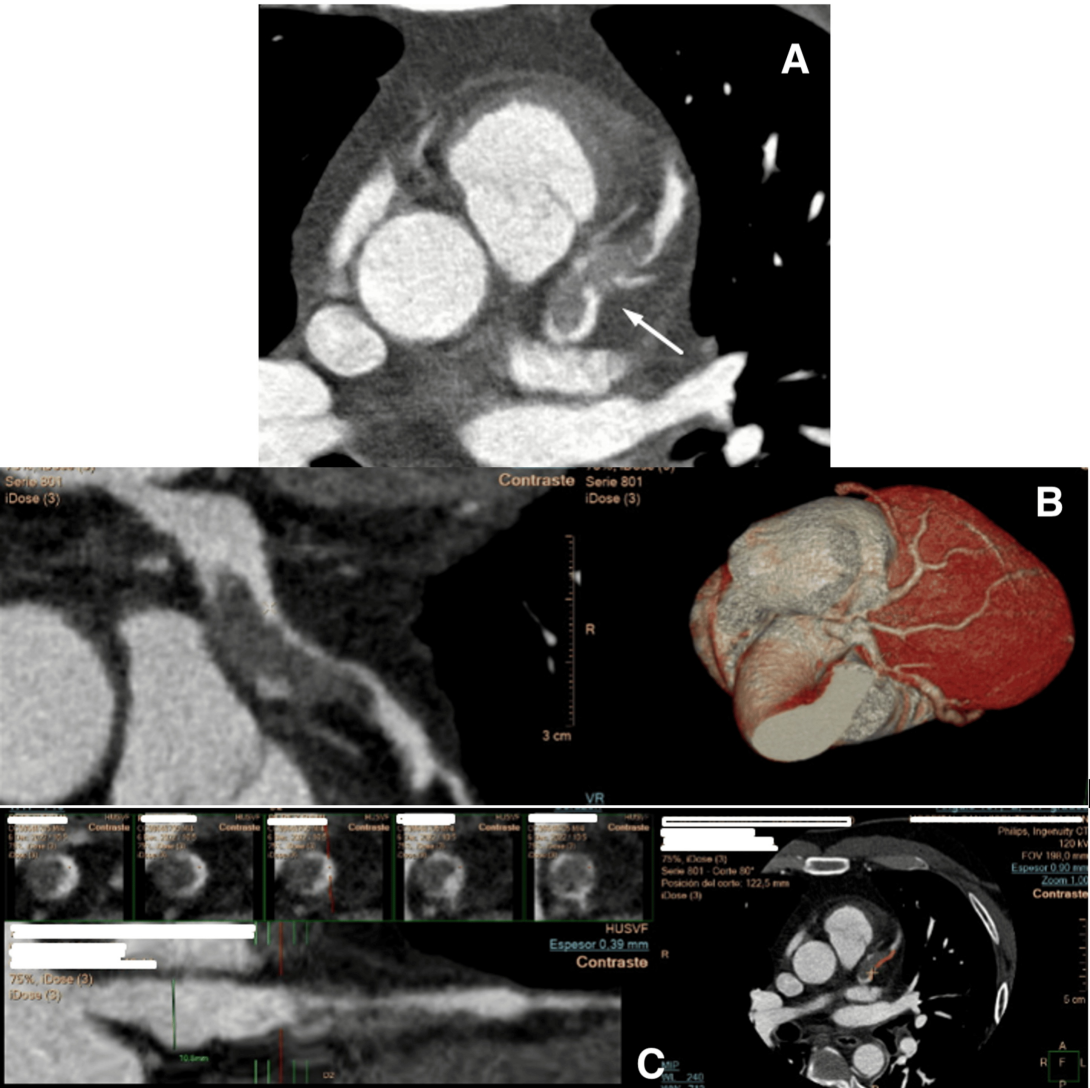
Coronary CT angiography showing a left main coronary artery aneurysm extending into the left anterior descending artery, with an acute intraluminal thrombus causing 80% luminal stenosis. (A) Axial CT angiographic image demonstrating aneurysmal dilatation of the left main coronary artery (arrow). (B) Curved multiplanar reconstruction of the left coronary artery combined with a 3D volume-rendered reconstruction, clearly depicting the aneurysm morphology and its extension into the left anterior descending artery. (C) Sequential cross-sectional views along the coronary lumen reveal the presence of intraluminal thrombus, responsible for approximately 80% luminal stenosis.

An aortogram (Figure 4) showed no evidence of thrombus or dissection; however, the LMCA demonstrated a large aneurysm with a filling defect suggestive of residual thrombus. Based on these findings, an emergent coronary CT angiography was ordered to rule out coronary artery dissection. The imaging revealed an LMCA aneurysm extending toward the LAD, measuring 40 mm in length and 20 mm in transverse diameter, with an acute intraluminal thrombus causing 80% stenosis and occlusion of the first septal branch (Figure 5).

**Figure 6:**
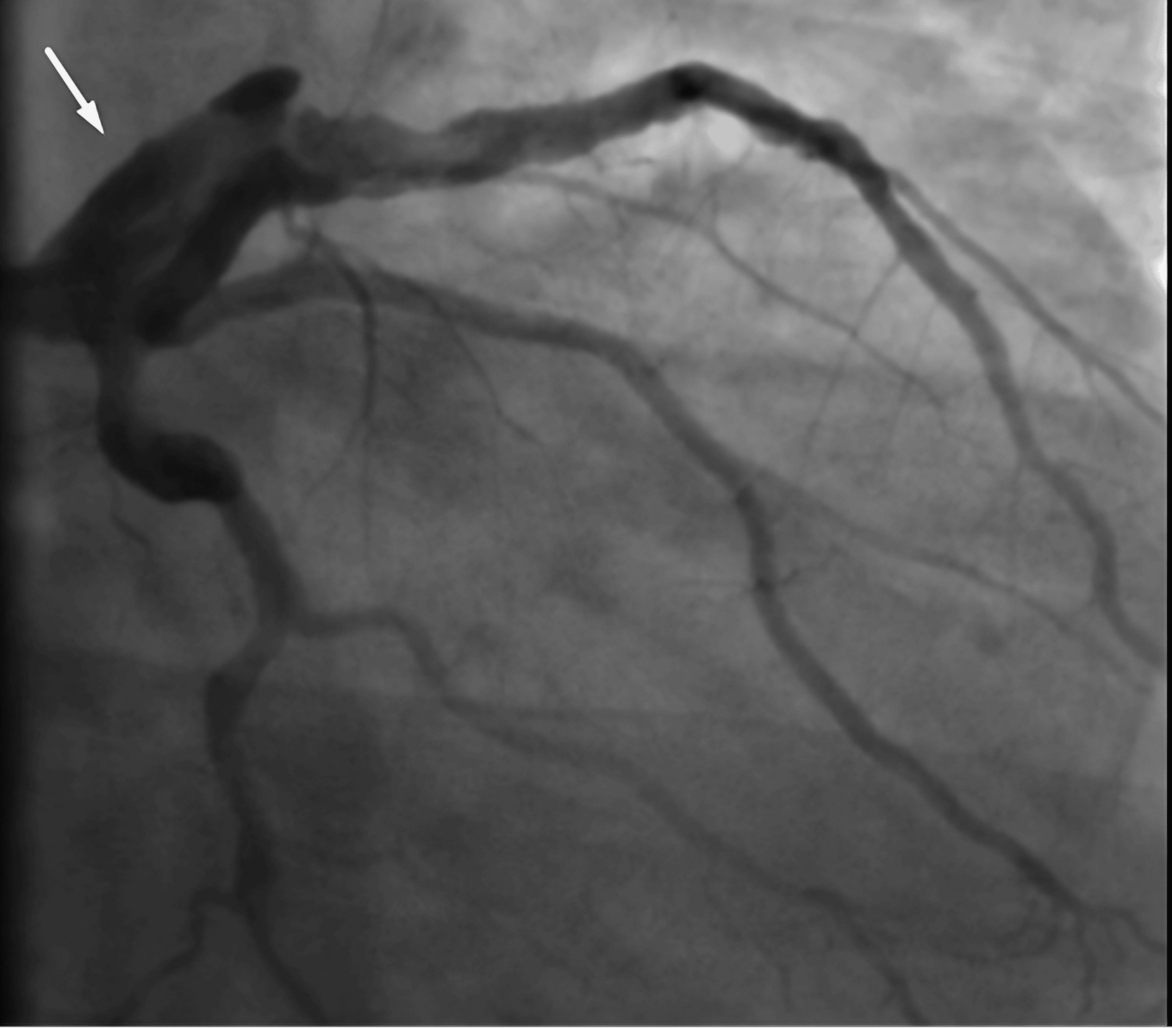
Follow-up coronary angiography at 72 hours shows a giant aneurysm of the left main coronary artery extending into the left anterior descending artery, with a high thrombus burden and poor response to tirofiban infusion. The arrow highlights the persistent aneurysmal dilation of the left main coronary artery at 72-hour follow-up, demonstrating a high thrombus burden despite tirofiban infusion.

The patient was evaluated for cardiovascular surgery but was not considered a surgical candidate due to recurrent thrombosis formation. He received tirofiban infusion and antithrombotic therapy for 72 hours. A repeat coronary angiography showed a persistent giant LMCA-to-LAD aneurysm with a high thrombus burden and poor response to tirofiban (Figure 6), without indication for percutaneous intervention.

A transthoracic echocardiogram showed akinesia of the apical and mid segments of the anterior wall and anterior septum, with an ejection fraction of 34% and a 1.5 × 5 mm apical thrombus. A multidisciplinary evaluation was performed to determine the etiology of the CAA, ruling out autoimmune, inflammatory, connective tissue, and infectious causes. Given the early paternal death, a congenital origin was considered the most likely.

The patient had an uneventful hospital course and was discharged on triple therapy with aspirin 100 mg, clopidogrel 75 mg, and warfarin 5 mg for one month, followed by indefinite therapy with clopidogrel and warfarin. At the six-month follow-up, he remained asymptomatic.

## Discussion

CAA disease is a relatively uncommon phenomenon. When located in the LMCA, these lesions are even rarer, occurring in approximately 3.5% of cases [1]. The most common etiology is atherosclerosis, followed by Kawasaki disease. Due to its low prevalence, there is no solid evidence on effective treatment strategies [2].

In the case presented, the involvement of the LMCA makes the decision difficult, whether medical treatment, percutaneous intervention, or surgery, given the limited information available. Coronary CT angiography provides essential information and, with coronary angiography, constitutes the imaging modalities of choice for evaluation. Most of the available evidence consists of case series and observational studies, as no randomized clinical trials are currently available.

Coronary aneurysms can cause myocardial ischemia depending on several factors, including size, location, and the presence of thrombus. The potential for complications such as acute coronary syndromes, ventricular dysfunction, and sudden cardiac death justifies the use of antithrombotic therapy in this setting.

A recent Chinese case series [5] described 19 patients with giant coronary aneurysms, mostly middle-aged men with atherosclerotic disease, about half of whom underwent surgical intervention. Among those managed medically, antithrombotic regimens varied: most received aspirin, approximately one-third were treated with dual antiplatelet therapy, and another third received anticoagulation, primarily with warfarin. Other case series report similar trends in antithrombotic use. In the same Chinese series, one patient was treated with direct oral anticoagulants (DOACs); however, despite their safety profile and proven efficacy in other settings, there is poor evidence for the use of DOACs in the context of coronary aneurysms. Given their larger size, giant CAAs are at higher risk of rupture, making anticoagulation decisions particularly complex and requiring careful individualized analysis. In the present case, the patient also had an additional indication for anticoagulation due to the presence of a left ventricular thrombus.

Another series has suggested that giant CAAs are associated with advanced age and a higher risk of complications, such as rupture, presentation as a cardiac or mediastinal mass, or superior vena cava syndrome [6]. Giant CAAs are also associated with coronary artery fistulas in approximately 25% of cases [7]. The International Coronary Artery Aneurysm Registry [8] described 1,561 patients with CAAs, 5.2% (82 patients) of whom had giant aneurysms, and 9.8% (8 patients) of these were located in the LMCA. While most patients in the registry underwent revascularization (68%), treatment strategies specific to giant CAAs were not detailed. Nevertheless, other series have shown that giant aneurysms are more often managed surgically [9]. Patients treated with percutaneous intervention are fewer, and the strategy and stent type are highly individualized. Covered stents (stent grafts), bare-metal stents, or coil embolization are the options considered [2]. Exclusive medical therapy is associated with a higher rate of complications and, overall, a worse prognosis [10].

In our case, despite the multiple risks, a conservative medical approach was chosen. This was supported by the initial benefit observed with thrombolysis during the acute phase. Technical challenges related to percutaneous revascularization included the aneurysm's size and location, the involvement of two coronary vessels, and a large myocardial territory at risk. Surgical intervention was also ruled out due to unfavorable anatomy, the presence of severe left ventricular dysfunction, and an apical thrombus. Despite additional management of full intravenous anticoagulation, the patient had a suboptimal response to tirofiban infusion, leaving indefinite anticoagulation therapy as the only viable option. Future advanced therapies, such as heart transplantation, may be considered depending on clinical progression.

## Conclusions

Patients with giant CAA represent a challenge for diagnostic and therapeutic interventions. These cases are often associated with a higher rate of complications, and surgical management is typically the preferred option, though percutaneous intervention may be performed in select cases.

Optimal antithrombotic therapy should ideally begin with dual antiplatelet therapy followed by a combination of an oral anticoagulant and a single antiplatelet agent. However, this strategy has not been validated in randomized clinical trials. No evidence currently supports the use of DOACs in this setting.

When located in the LMCA, CAAs likely represent the highest-risk scenario due to the technical challenges associated with revascularization. Based on case reports and observational studies, management should be individualized, as the overall prognosis remains poor.
